# Inhibitory Effects of Grape Stem Extracts From Douro Varieties on Skin‐Aging Enzymes: A Sustainable Source of Cosmeceutical Agents

**DOI:** 10.1111/jocd.70762

**Published:** 2026-03-19

**Authors:** Maria Garcia‐Marti, Oumaima Boutaub, Amanda Priscila Silva Nascimento, Jesus Simal‐Gandara, Ana Novo Barros

**Affiliations:** ^1^ Departamento de Química Analítica e Alimentaria, Facultade de Ciencias Universidade de Vigo Ourense Spain; ^2^ Centre for Research and Technology of Agro‐Environmental and Biological Sciences (CITAB) University of Trás‐os‐Montes and Alto Douro (UTAD) Vila Real Portugal; ^3^ Academic Unit of Food Engineering Federal University of Campina Grande Campina Grande PB Brazil

**Keywords:** cosmetic, grape stems, phenolic profile, sustainability, valorization

## Abstract

**Background:**

Grape stems are a major by‐product of the winemaking process and remain largely underexploited despite their richness in bioactive phenolic compounds, representing an opportunity for sustainable valorization within a circular economy framework.

**Aims:**

This study aimed to characterize the phenolic composition and evaluate the antioxidant and enzyme inhibitory activities of grape stem extracts from different cultivars grown in the Douro Demarcated Region.

**Methods:**

Grape stems from 12 *Vitis vinifera* cultivars were extracted and analyzed for total phenolic content, ortho‐diphenols, and flavonoids. Antioxidant capacity was assessed using FRAP and DPPH assays, while bioactivity was further evaluated through elastase and hyaluronidase inhibition assays.

**Results:**

Total phenolic content ranged from 35.2 to 89.6 mg GAE/g DW, with Sousão exhibiting the highest values. Ortho‐diphenols and flavonoids showed marked cultivar‐dependent variability, with Verdelho and Cercial presenting values above 20 mg CAE/g DW and 15 mg QE/g DW, respectively. Antioxidant activity reached up to 920 μmol TE/g DW (FRAP) and 85% DPPH radical scavenging. Enzyme inhibition assays revealed strong cultivar‐dependent bioactivity, with Sousão, Verdelho, and Gouveio extracts achieving over 60% inhibition of elastase and hyaluronidase.

**Conclusions:**

The results highlight the significant influence of grape cultivar on the bioactive potential of grape stems and support their valorization as promising raw materials for nutraceutical and cosmeceutical applications.

## 1 Introduction

Winery by‐products have traditionally been discarded, causing waste of resources and environmental issues [1]. These by‐products represent approximately 20%–30% of the total grapes processed during winemaking, equivalent to approximately 20 million tons generated annually [2]. Although wine grape production has slightly declined in recent years [3], the seasonal nature of wine grape residue generation leads to high concentrations of these residues accumulating in specific areas and periods, causing environmental impacts if not managed sustainably [4]. Therefore, revaluing these winery wastes is essential to transform them into high‐value products and enhance the circular economy to align with the United Nations Sustainable Development Goals and reduce food waste along production and supply chains [5].

By‐products generated during the winemaking process include matrices as diverse as stems, skins, seeds, pomace, and lees [6]. The yield and phytochemical characteristics of these wastes vary significantly depending on internal factors such as the grape variety used and its ripening stage [7], as well as the environmental conditions [8] and technology employed during harvesting and winemaking [9]. In this sense, winery by‐products have been widely described as a relevant source of bioactive molecules, being phenolic compounds the most widely distributed secondary metabolites found in grapes and their derivatives [10]. Phenolic compounds, which exhibit a wide range of physiological properties [11], find applications in food [12], pharmacology [13], and cosmetics [14]. Although most articles in the literature focus on the extraction and application of these compounds from by‐products such as grape pomace or skins, there is a gap in studies using grape stems for this purpose, despite evidence that they are also a potential source of these phytochemicals [15, 16]. Grape stems constitutes the skeleton of grape brunch, representing between 3% and 6% of their total weight [17]. This waste, obtained during the destemming process in winemaking, have traditionally been used as fertilizer [18] or animal feed [19]. However, there is an increasing interest on the upcycling and valorization of this wine side streams into alternative applications [20]. Thus, recent studies conducted by our research group have demonstrated that phenolic compounds present in grape stems possess antioxidant [21], antimicrobial [22, 23], and anti‐aging properties [24, 25]. The bioactive characteristics of phenolic compounds hold promise for preventing and treating different issues associated with aging [26]. In recent decades, more attention has been devoted to skin care to maintain a favorable esthetic appearance and to prevent many dermatological disorders that may affect general health condition [27]. Skin aging is characterized by a loss of elasticity and volume, as well as uneven pigmentation resulted from multiple interconnected processes involving endogenous stressors as oxidative stress and external stimuli as UV irradiation [28]. Polyphenols possess the ability to act as biological antioxidants and activate cell signaling pathways to induce the expression of antioxidant enzymes [29], while inhibiting anti‐aging molecules such as elastase and hyaluronidase, and depigmenting activity as tyrosinase [30]. These reactions occurs thanks to the presence of the hydroxyl group and the phenyl group in the phenolic ring, as well as the interaction of their respective phenyl ring with the oxygen, carbon and organic acid molecule [31]. Consequently, polyphenols extracted from natural sources provide numerous health benefits, including mitigating effects on skin aging [32]. By influencing the molecular pathways involved in senescence, the bioactive properties of polyphenols can help prevent or delay their activation, thereby reducing or alleviating aging and age‐related skin conditions [33].

Based on the previous findings, the aim of this research was to investigate the phenolic and antioxidant profile of grape stems derived from the winemaking process of 12 different red varieties from the *Região Demarcada do Douro*, Portugal. This approach consisted of analyzing total phenolic content (TPC), *ortho*‐diphenol content (ODC), and flavonoid content (FP) using spectrophotometric assays, followed by tentative identification of these compounds using high‐performance liquid chromatography (HPLC) coupled to a photodiode array. Subsequently, the antioxidant capacity of the studied extracts was also analyzed using two radical scavenging methods: 2,2‐azino‐bis (3‐ethylbenzothiazoline‐6‐sulfonic acid) diammonium salt (ABTS) and 2,2‐diphenyl‐1‐picrylhydrazyl (DPPH) and using the ferric antioxidant/reducing power (FRAP) assay. Finally, the effect of these bioactive compounds on key enzymes involved in skin aging, namely elastase, hyaluronidase, and depigmenting activity as tyrosinase, were also determined. The results of this study will serve as a foundation for exploring the potential revalorization of these by‐products from the wine industry for cosmetic applications.

## 2 Materials and Methods

### 2.1 Sampling

Grape stems (engraços) from 12 *Vitis vinifera* L. cultivars were collected during the 2024 harvest from the Douro Demarcated Region, specifically within the Douro Superior sub‐region, Portugal. The selection included five red grape varieties (Tinta Francisca, Tinta Roriz, Tinta Amarela, Sousão, and Rufete) and seven white varieties (Rabigato, Gouveio, Síria, Códega do Larinho, Verdelho, Cercial, and Folgasão), commonly used in the region's winemaking practices. Immediately after the destemming process, the fresh stems were rinsed with distilled water to remove residual must and impurities. The clean material was then oven‐dried at 40°C for 72 h in a ventilated drying chamber to preserve thermolabile compounds. Once dried, the samples were ground to a fine powder using a blade, and the resulting powders were stored in vacuum‐sealed bags, protected from light and humidity, at room temperature until further analysis.

### 2.2 Phenolic Compounds Extraction Process

Phenolic compound extraction was carried out by weighing 40 mg of the pulverized grape stems sample into Eppendorf vials. Then, 1.5 mL of a mixture of 70% ethanol and distilled water in a 70:30 (v/v) ratio was added. The samples were shaken in a circular motion, then transferred to a rotary shaker at room temperature for half an hour and centrifuged at 5000 rpm for 15 min at 4°C (Sigma 2‐16 K centrifuges, Germany) to obtain a supernatant, which was poured into a 5 mL volumetric flask. This process was repeated three times, and the final volume was adjusted with a mixture of 70% ethanol and distilled water (70:30, v/v).

### 2.3 Determination of Phenolic Content

The phenolic content of the stem extracts was determined using spectrophotometric methodologies adapted for 96‐well microplates (PrimeSurface MS‐9096MZ, Frilabo, Maia, Portugal), according to Breda et al. [34], with some modifications. Finally, the absorbances were measured using microplate readers (Multiskan GO Microplate Photometer, Thermo Fisher Scientific, Vantaa, Finland).

#### 2.3.1 Total Phenols Content

The total phenols content of grape stems extracts was determined by adding 20 μL of the sample to 100 μL of Folin–Ciocalteu reagent previously diluted with water (1:10 H_2_O). Then, 80 μL of Na_2_CO_3_ (7.5%) was added. The reaction was incubated in an oven at 40°C–45°C for 30 min and protected from light. Absorbance was measured at 750 nm. Gallic acid was used as a standard, and the results are expressed in mg of gallic acid per gram of dry weight (mg GA/g DW) using gallic acid as standard.

#### 2.3.2 *Ortho*‐Diphenols Content

The *ortho*‐diphenols content of grape stems extracts was determined by adding 40 μL of Na_2_MoO_4_ (50 g/L) to 160 μL of the samples appropriately diluted. Mixtures were vortexed and allowed to rest at room temperature, protected from light, for 15 min. The absorbance was measured at 375 nm and quantified using gallic acid as standard. Results are expressed in mg GA/g DW.

#### 2.3.3 Flavonoids Content

The flavonoid content of sample extracts was measured based on the formation of a flavonoid–aluminum complex. Firstly, 24 μL of the diluted sample was mixed with 28 μL of NaNO_2_ (50 g/L). After exactly 5 min, 28 μL AlCl_3_ (100 g/L) was added, and the mixture was allowed to react for 6 min. Finally, 120 μL of NaOH (1 M) was added to the mixture. The absorbance was immediately measured at 510 nm. Catechin was used for the construction of the calibration curve. Results are expressed in mg of catechin per gram of dry weight (mg CAT/g DW).

### 2.4 Antioxidant Capacity Assays

Antioxidant capacity was determined using three different assays: DPPH, ABTS, and FRAP, according to the methodology described by Branco et al. [35]. These analyses were performed in triplicate (*n* = 3) for each sample, standard, and blank. The analysis was performed using 96‐well microplates (Frilabo, Milheirós, Portugal) with absorbance readings measured using a MultisKan FC microplate reader (Thermo Fisher Scientific, Lisboa, Portugal).

#### 2.4.1 DPPH Method

To assess the antioxidant capacity by DPPH method, 35 mg of DPPH reactive were dissolved in 10 mL of methanol to create a DPPH solution. The solution was subsequently mixed with an ethanol/water blend (70:30, v/v) to reach an absorbance of around 1.000 at 520 nm. Afterwards, 190 μL of this solution were added to each well along with 10 μL of Trolox standard, sample, or blank (ethanol/water, 70:30 (v/v)). Absorbance at 520 nm was measured following a 30‐min incubation period. The radical scavenging activity was calculated as the percentage of inhibition, accordling to Equation (1):

$$\%\text{Inhibition}=\left[\left(\mathrm{ABS}520_{\text{BLANK}}-\mathrm{ABS}520_{\text{SAMPLE}}\right)/\mathrm{ABS}520_{\text{BLANK}}\right]\times 100\;\;\;\;\;\;\;\;\;\;\;(1)$$

Findings were quantified in mmol of Trolox equivalents per hundred grams of dried sample (mmol Trolox/100 g dry weight (dw)), using a Trolox standard curve that ranged from 0.039 to 1.25 mM.

#### 2.4.2 ABTS (2,2′‐Azino‐Bis‐(3‐Ethylbenzothiazoline‐6‐Sulfonic Acid)) Method

Antioxidants cause the ABTS radical cation (${\text{ABTS}}^{⦁+}$) to change from blue/green to colorless. 7 mM ABTS was mixed with 88 μL of 140 mM potassium persulfate ($K_{2}O_{8}S_{2}$), and was incubate in darkness at room temperature for 12–16 h. Then, the ABTS radical cation was diluted with sodium acetate buffer (${\mathrm{CH}}_{3}{\mathrm{COO}}^{-}/{\mathrm{Na}}^{+}$, 100 mM, pH 4.5) to an absorbance of 0.70 ± 0.02 at 734 nm to create the ABTS working solution. A calibration curve was constructed using Trolox as standard (ranging from 0.034 to 0.2 mM). To assess the percentage of inhibition 12 μL of each standard solution and sample were added to each well followed by 188 μL of ABTS working solution. In addition, three wells containing 12 μL of distilled water and 188 μL of ABTS working solution were used as blank. The calculation for inhibition percentage was determined in Equation 2:

$$\%\text{Inhibition}=\left[\left(\mathrm{ABS}734_{\text{BLANK}}-\mathrm{ABS}734_{\text{SAMPLE}}\right)⁄\mathrm{ABS}734_{\text{BLANK}}\right]\times 100\;\;\;\;\;\;(2)$$

The antioxidant capacity was expressed in mmol of Trolox per hundred grams of sample (mmol Trolox/100 g).

#### 2.4.3 FRAP (Ferric Reducing Antioxidant Power) Method

The antioxidant capacity using the FRAP assay, involves reducing ${\mathrm{Fe}}^{3+}$ to ${\mathrm{Fe}}^{2+}$ present in the FRAP reagent, forming a blue complex with TPTZ (2,4,6‐Tris(2‐pyridyl)‐s‐triazine) that is then measured at 593 nm. A 5 mM Trolox standard solution was serially diluted to prepare concentrations ranging from 0.039 to 1.25 mM. 20 μL of sample or standard was combined with 180 μL of FRAP working solution (made from 20 mL of 300 mM acetate buffer (pH 3.6), 2 mL of 10 mM TPTZ (in 40 mM HCl), and 2 mL of 20 mM ferric chloride in water) in every well of the microplate. Afterwards, FRAP working solution was prepared and then heated at 37°C for 10 min before use. The blends were agitated and left to sit at 37°C in the absence of light for 30 min. Results were expressed in mmol of Trolox per hundred grams of sample (mmol Trolox/100 g dw).

### 2.5 HPLC–DAD–ESI–MS/MS Analysis of the Quantitative (Poly)phenolic Profile of Grape Stems

Following the methodology proposed by Vieira et al. [36], samples (100 mg) were extracted with 1.5 mL of a mixture containing ethanol and distilled water (70:30, v/v, acidified with formic acid 0.1%). For the identification and quantification of the phenolic compounds, chromatographic separations were carried out using a Thermoscientific VANQUISH C18 column (150.1 mm, 2.2 μM particle size; Thermo Fisher Scientific Inc., Lithuania). The chromatographic resolution of the phenolic profile was achieved using deionized water/formic acid (99.9:0.1, v/v) (A) and acetonitrile/formic acid (99.9:0.1, v/v) (B) as chromatographic solvents using the following gradient (Time, %B): (0, 10%), (20, 60%), (20.1, 10%), and (25, 10%). The flow rate was 0.3 mL/min, and the injection volumes were 7 μL. The HPLC system was equipped with a Vanquish—LTQ‐XL—ThermoScientific diode array and a mass detector in series (Thermo Scientific Dionex UltiMate 3000 Series, Germany). It consisted of a quaternary SD, RS, BM, and BX pump, a WPS‐3000 autosampler, a G1322A degasser, and an Electrochemical photodiode array detector controlled by Xcalibur software version 08.03 (Agilent Technologies, Waldbronn, Germany). Spectroscopic data from all peaks were accumulated in the range of 240–600 nm, and the spectral data were recorded at 280, 330, and 370. The mass detector was a G2445A Ion‐Trap Mass Spectrometer equipped with an electrospray ionization (ESI) system and controlled by LTQ Tune software version 4.1 (Agilent, Waldbronn, Germany). Nitrogen was used as a nebulizing gas at a pressure of 60 psi, and the flow was adjusted to 11 L/min. The heated capillary and voltage for ionization were maintained at 350 C and 5 kV, respectively. Collision‐induced fragmentation experiments were performed in the ion trap using helium as a collision gas, with voltage ramping cycles from 0.3 up to 2 V. The full scan mass covered the range from m/z 100 up to m/z 2000. Mass spectrometry data were acquired in the negative ionization mode. Total ion chromatograms were recorded as full scan mass spectra (MS). The identification of the individual phenolic compounds was performed by analyzing the retention time (min), parent ions, and fragmentation patterns in comparison with authentic standards and, when they were not available, descriptions available in the literature. Phenolic compounds were quantified by DAD chromatograms recorded at the abovementioned wavelengths, using freshly prepared calibration curves of 5‐*O*‐caffeoylquinic acid for phenolic acids (*r* ^2^ = 0.9995), epicatechin for flavan‐3‐ols (*r* ^2^ = 1.0000), quercetin‐3‐*O*‐rutinoside for flavonols (*r* ^2^ = 0.9999), and resveratrol for stilbens (*r* ^2^ = 0.9996) for each day of analysis.

### 2.6 Anti‐Aging and Skin Depigmenting Activity

The inhibition of elastase and hyaluronidase enzymes was measured to evaluate anti‐aging activity. For this analysis, the extract was obtained using a rotative evaporator (Bibby Scientific Limited, Stone, Staffordshire, UK) and then freeze‐dried in BenchTop Pro with omnitronics, SP Scientific, Warminster, PA, USA. Following Serra et al. [37], 1 mg of hydro methanolic extract (70:30, v/v) was dissolved in 1 mL of 10% DMSO.

#### 2.6.1 Elastase Inhibition

To determine elastase inhibition, 50 μL of grape stem extracts (dissolved in 10% DMSO at 1 mg/mL) or negative control (Tris–HCl buffer 0.2 mM, pH 8) was added to 160 μL of buffer in 96‐well plates. Then, 20 μL of N‐Succinyl‐Ala‐Ala‐Ala‐p‐nitroanilide (substrate) was added to the mixture. Afterwards, the mixture was incubated at room temperature, protected from light, for 10 min. Finally, 20 μL of elastase (1 U/mL) was added, and the absorbance was measured at 410 nm using a microplate reader. This assay was calculated according to the following Equation (3):

$$\text{Elastase inhibition}\;\left(\%\right)=\left[\left(\mathrm{Abs}410\;\text{control}-\mathrm{Abs}410\;\text{sample}\right)/\left(\mathrm{Abs}410\;\text{control}\right)\right]\times 100\;\;\;\;\;\;\;\;\;\;\left(3\right)$$

#### 2.6.2 Hyaluronidase Inhibition

For the hyaluronidase inhibition assay, 140 μL of Tris–HCl buffer (50 mM, pH 7.0) was initially mixed with 20 μL of hyaluronidase (4 mg/mL), 20 μL of hyaluronic acid sodium salt (0.4 mg/mL), and 20 μL of the grape stem extracts or 20 μL of Tris–HCl buffer as the negative control. The mixture was then incubated at 37°C for 60 min. After incubation, the microplate was left to cool to room temperature. Finally, 20 μL of cetylpyridinium chloride solution (10%) was added and the absorbance was measured at 600 nm using a microplate reader. This enzymatic inhibition (%) was calculated according to the following Equation (4):

$$\text{Hyaluronidase inhibition}\;\left(\%\right)=\left[\left(\mathrm{Abs}600\;\text{control}-\mathrm{Abs}600\;\text{sample}\right)/\left(\mathrm{Abs}600\;\text{control}\right)\right]\times 100\;\;\;\;\;\;\;\;\;\left(4\right)$$

#### 2.6.3 Tyrosinase Inhibition

To assess tyrosinase inhibition, 10 μL of grape stem extract, 10 μL of 10% DMSO (negative control), or 10 μL of Kojic acid (positive control) was added to a microplate. Subsequently, 20 μL of tyrosinase (1000 U/mL) and 170 μL of a mixture containing l‐tyrosine solution, phosphate buffer (50 mM, pH 6.5), and distilled water in a 10:10:9 ratio were added. The mixtures were incubated at 37°C for 10 min, after which the absorbance was measured at 490 nm using a microplate reader. This assay was calculated according to the following Equation (5):

(5) $$\text{Tyrosinase inhibition}\;\left(\%\right)=\left[\left(\mathrm{Abs}490\;\text{control}-\mathrm{Abs}490\;\text{sample}\right)/\left(\mathrm{Abs}490\;\text{control}\right)\right]\times 100$$

### 2.7 Statistical Analysis

All statistical analyses were performed using SPSS software, version 29.0. The data were first checked for normality using the Shapiro–Wilk test and for homogeneity of variances using Levene's test. Parameters that met the assumptions of normality and homogeneity of variances were compared between the two apple pomaces using Student's *t*‐test. For multiple comparisons, Tukey's Honest Significant Difference (HSD) test was applied as a post hoc analysis to identify specific differences between the groups. The level of statistical significance was set at *p* < 0.05. All results are presented as means ± standard deviations. If the assumptions of normality or homogeneity of variance were violated, appropriate nonparametric tests were applied (e.g., Mann–Whitney *U* test) and were noted accordingly.

## 3 Results and Discussion

### 3.1 Phenolic Content

The quantitative evaluation of phenolic compounds in grape stem extracts revealed significant varietal differences across total phenolics (TPC), ortho‐diphenols (ODC), and flavonoid content (FC) (Table 1).

**TABLE 1:** Total phenolic content (TPC), ortho‐diphenols (ODC), and flavonoid content (FC) in grape stem extracts from 12 Douro cultivars.

Grape Stem	TPC (mg GAeq/g dw)	ODC (mg GAeq/g dw)	FC (mg CATeq/g dw)
Tinta Amarela	87.04 ± 4.71^c,d^	57.48 ± 2.58^e^	50.63 ± 1.66^e^
Tinta Roriz	58.46 ± 0.92^g^	49.69 ± 1.95^f^	43.73 ± 3.81^f^
Sousão	131.53 ± 2.51^a^	114.12 ± 1.67^a^	122.27 ± 0.57^a^
Tinta Francisca	89.85 ± 1.69^c,d^	53.41 ± 4.67^e,f^	37.52 ± 0.83^g^
Rufete	103.82 ± 4.30^b^	63.44 ± 0.60^d,e^	75.30 ± 2.89^c,d^
Folgasão	69.76 ± 4.39^f^	49.68 ± 2.17^f^	46.20 ± 3.21^e,f^
Síria	82.17 ± 2.37^d,e^	63.23 ± 0.66^d,e^	90.41 ± 5.27^b^
Rabigato	85.53 ± 1.87^d,e^	65.83 ± 1.84^d^	47.90 ± 4.62^e^
Gouveio	90.84 ± 1.22^c,d^	67.64 ± 1.77^d^	85.75 ± 1.86^b,c^
Verdelho	102.96 ± 3.05^b^	72.18 ± 1.94^c^	73.88 ± 4.28^d^
Códega de Larinho	95.21 ± 3.08^b,c^	65.25 ± 4.16^d^	40.34 ± 1.06^f,g^
Cercial	86.35 ± 3.11^d,e^	89.83 ± 2.56^b^	77.33 ± 1.44^c,d^

*Note:* Results are expressed as mean ± SD (*n* = 6). Different letters within the same column indicate statistically significant differences among cultivars (*p* < 0.05), according to one‐way ANOVA followed by Tukey's post hoc test.

Abbreviations: FC, flavonoid content; ODC, ortho‐diphenols; SD, standard deviation; TPC, total phenolic content.

Among the cultivars tested, Sousão consistently exhibited the highest levels in all three parameters: 131.53 ± 2.51 mg GAeq/g dw for TPC, 114.12 ± 1.67 mg GAeq/g dw for ODC, and 122.27 ± 0.57 mg CATeq/g dw for FC. These results indicate that Sousão grape stems are a particularly rich matrix of bioactive phenolic compounds, including structurally complex and highly active ortho‐substituted derivatives.

Other cultivars, such as Rufete, Verdelho, and Cercial, also presented notably high values of TPC and flavonoids, although significantly lower than Sousão (*p* < 0.05). Rufete, for instance, had TPC of 103.82 ± 4.30 mg GAeq/g dw and FC of 75.30 ± 2.89 mg CATeq/g dw, reflecting a well‐balanced phenolic profile. Cercial stood out due to its elevated ODC (89.83 ± 2.56 mg GAeq/g dw), despite presenting moderate TPC and FC, which suggests a possible enrichment in specific ortho‐diphenolic compounds such as hydroxytyrosol or certain flavonols with catechol‐type substitution.

In contrast, cultivars such as Tinta Roriz, Tinta Francisca, and Codega do Larinho displayed the lowest values across the three parameters. Tinta Francisca, for example, recorded only 37.52 ± 0.83 mg CATeq/g dw in flavonoid content, while Tinta Roriz had the lowest TPC (58.46 ± 0.92 mg GAeq/g dw) and among the lowest ODC levels (49.69 ± 1.95 mg GAeq/g dw), suggesting a limited presence of both total and ortho‐phenolic structures in its stems.

Statistical analysis using one‐way ANOVA followed by Tukey's post hoc test (*p* < 0.05) confirmed significant differences among cultivars for all three parameters. These data reinforce the influence of genetic background on the phenolic metabolism of grapevines, even in residual tissues such as stems, and highlight the potential of varietal selection for targeted applications in food, nutraceutical, or cosmetic formulations. Specifically, cultivars rich in ortho‐diphenols and flavonoids—such as Sousão, Síria, and Gouveio—are of particular interest due to the known antioxidant, metal‐chelating, and anti‐inflammatory properties of these compounds.

Recent investigations have profiled the phenolic composition of grape stems from Portuguese and Mediterranean cultivars, emphasizing the relevance of varietal and regional factors. In the present study, total phenolic content (TPC), *ortho*‐diphenols (ODC), and flavonoid content (FC) were quantified in grape stems from 12 cultivars harvested in 2024 in the Douro Superior sub‐region, under similar climatic and edaphic conditions. All values are expressed on a dry weight basis and were obtained using standardized spectrophotometric methods with hydroethanolic extraction (EtOH:H_2_O, 50:50 v/v) [38, 39].

According to previous literature, TPC values in grape stems typically range between 90 and 110 mg GAeq/g dw, depending on genotype, growing conditions, and harvest year [40]. In our study, Sousão presented the highest TPC (131.53 ± 2.51 mg GAeq/g dw), exceeding reported ranges for Mediterranean cultivars such as Garnacha, Tempranillo, or Mazuelo [40]. Other cultivars, including Rufete (103.82 ± 4.30) and Verdelho (102.96 ± 3.05), aligned with previous data, while Tinta Roriz displayed a significantly lower TPC (58.46 ± 0.92), suggesting a strong varietal effect on phenolic content. Regarding *ortho*‐diphenols (ODC), previously published values span from 50 to 80 mg GAeq/g dw across both red and white grape varieties [41, 42]. Here, Sousão again exhibited the highest ODC (114.12 ± 1.67), followed by Cercial (89.83 ± 2.56). The latter result is particularly noteworthy, as this cultivar presented moderate TPC and FC levels, indicating a possible selective accumulation of ortho‐substituted phenolics, such as catechol derivatives. This pattern has been observed in other cultivars where catechin‐type compounds predominate [42].

As for flavonoid content (FC), literature reports values between 50 and 100 mg CATeq/g dw, with some regional blends averaging around 70 mg/g [24, 25, 40]. In this context, Síria (90.41 ± 5.27) and Gouveio (85.75 ± 1.86) are within the expected range, while Sousão once again stood out with an elevated FC (122.27 ± 0.57), suggesting consistently high levels of flavonoid compounds, possibly linked to environmental stress conditions or genotype‐driven expression of the flavonoid biosynthetic pathway.

All samples analyzed originated from the Douro Superior during the 2024 vintage, a factor that provides coherence to the comparative framework. Although climate and soil type were consistent across samples, notable differences in phenolic profiles were observed among cultivars. These differences are likely attributed to genetic background and specific phenolic biosynthetic capacities.

Several cultivars—particularly Sousão and Cercial—showed phenolic profiles that exceed previously established baselines for grape stems from both Iberian and Central European cultivars. These findings support the importance of varietal selection in upcycling grapevine by‐products and highlight the potential of these stems as a rich source of specific phenolic subclasses, especially *ortho*‐diphenols and flavonoids. Future work using chromatographic and mass spectrometry techniques is needed to elucidate the molecular composition and possible applications in food, nutraceuticals, or cosmetics.

When phenolic parameters were jointly interpreted, cultivars could be qualitatively grouped according to their overall phenolic profiles. Sousão stood out due to its simultaneously high TPC, ODC, and FC values, while cultivars such as Gouveio, Síria, and Verdelho exhibited intermediate but balanced phenolic compositions. In contrast, Tinta Roriz and Códega de Larinho consistently showed lower phenolic levels across all parameters.

### 3.2 Antioxidant Capacity

The antioxidant capacity of grape stem extracts from 12 *Vitis vinifera* cultivars was assessed using three complementary in vitro assays: ABTS and DPPH radical scavenging capacity, and the ferric reducing antioxidant power (FRAP). All results were expressed as Trolox equivalents (mmol Trolox/g dry weight), providing standardized comparability across methods. Statistically significant differences were observed among cultivars (*p* < 0.05), highlighting a strong genotype‐dependent effect (Table 2).

**TABLE 2:** Antioxidant capacity of grape stem extracts from 12 Douro cultivars assessed by ABTS, DPPH, and FRAP assays.

Grape stem	ABTS	DPPH	FRAP
	(mmol Trolox/g)	(mmol Trolox/g)	(mmol Trolox/g)
	IC_50_ (mean ± SD)	IC_50_ (mean ± SD)	
Tinta Amarela	0.158 ± 0.001^e^	1.374 ± 0.223^f^	0.2754 ± 0.0120^c^
Tinta Roriz	0.236 ± 0.005^c^	3.288 ± 0.540^a^	0.2771 ± 0.0263^b^
Sousão	0.099 ± 0.001^i^	0.920 ± 0.012^h^	0.5678 ± 0.0004^a^
Tinta Francisca	0.136 ± 0.001^f^	1.748 ± 0.357^d^	0.0843 ± 0.00533^l^
Rufete	0.216 ± 0.003^d^	1.816 ± 0.444^c^	0.0934 ± 0.0023^k^
Folgasão	0.113 ± 0.002^g^	2.259 ± 0.016^b^	0.2615 ± 0.0043^d^
Síria	0.336 ± 0.011^a^	2.259 ± 0.016^b^	0.1456 ± 0.0053^h^
Rabigato	0.123 ± 0.0010^g^	1.166 ± 0.009^g^	0.0989 ± 0.0032^k^
Gouveio	0.108 ± 0.0010^h^	1.921 ± 0.005^c^	0.2276 ± 0.0084^e^
Verdelho	0.097 ± 0.0015^j^	1.078 ± 0.0493^g^	0.1219 ± 0.0011^j^
Códega de Larinho	0.101 ± 0.0015^h^	1.351 ± 0.017^f^	0.1876 ± 0.0088^f^
Cercial	0.255 ± 0.0010^b^	1.540 ± 0.095^e^	0.3136 ± 0.0143^a^

*Note:* Results are expressed as mean ± SD (*n* = 3). Different letters within the same column indicate statistically significant differences among cultivars (*p* < 0.05), according to one‐way ANOVA followed by Tukey's post hoc test.

Abbreviations: ABTS, scavenging capacity of the ABTS• + radical; DPPH, scavenging capacity of the DPPH• radical; FRAP, ferric reducing antioxidant power; SD, standard deviation.

Among all cultivars, Sousão and Verdelho exhibited the highest antioxidant capacities. The extract from Sousão showed the lowest IC_50_ values in both ABTS (0.099 mg/mL) and DPPH (0.920 mmol Trolox/g dw), and the highest FRAP value (0.568 mmol Trolox/g dw). Verdelho also presented consistently strong antioxidant performance (ABTS: 0.097 mmol Trolox/g dw; DPPH: 1.078 mmol Trolox/g dw; FRAP: 0.314 mmol Trolox/g dw). These findings are in line with recent studies showing that catechin‐ and epicatechin‐rich grape stem extracts display high radical scavenging and reducing capacity when standardized against Trolox [24, 25, 43].

In contrast, Tinta Roriz and Síria were the least active cultivars, with Tinta Roriz exhibiting the highest IC_50_ in the DPPH assay (3.288 mmol Trolox/g dw) and Síria showing the highest IC_50_ in ABTS (0.336 mmol Trolox/g dw) and one of the lowest FRAP values (0.146 mmol Trolox/g dw). These results are consistent with previous reports indicating limited phenolic content and antioxidant capacity in stems of these varieties [22, 23, 44].

While the ABTS and DPPH assays evaluate free radical scavenging through hydrogen or electron transfer, the FRAP assay quantifies reducing capacity via electron donation. Despite these mechanistic differences, cultivars such as Sousão, Verdelho, and Folgasão consistently ranked among the top in all three assays. This agreement reinforces their high antioxidant potential and supports their valorization for health‐related applications. Similar inter‐method consistency has been reported by Blackford et al. [45], who found strong correlations between ABTS, DPPH, and FRAP assays in Trolox‐standardized grape stem extracts [46].

Of particular interest is the high antioxidant capacity exhibited by several white cultivars, including Verdelho, Rabigato, and Gouveio, which rivaled or outperformed some red varieties. This challenges the common assumption that red grape residues are necessarily richer in bioactive compounds and aligns with recent literature that underscores the functional potential of white grape by‐products [45, 47].

In summary, the results confirm the substantial antioxidant variability among grape stem extracts from different cultivars and highlight the potential of specific genotypes, especially Sousão and Verdelho, as valuable raw materials for the development of functional ingredients or nutraceuticals. Standardization against Trolox provides reliable cross‐study comparability and should be adopted as a consistent practice in future antioxidant evaluations.

Although in vitro assays such as ABTS, DPPH, and FRAP do not fully reproduce physiological conditions, the convergence of results across methods strengthens the reliability of these findings. The high activity of Sousão and Verdelho can be directly linked to their elevated levels of proanthocyanidins and flavonols (see Section 3.3), reinforcing the role of specific phenolic subclasses in determining antioxidant potential. Reported ranges for grape stems from Spanish and Brazilian cultivars situate these Douro varieties among the highest performers [38, 41]. This not only supports their valorization as antioxidant‐rich by‐products but also highlights the industrial relevance of selecting varieties with stable antioxidant profiles for food and cosmetic formulations.

A joint interpretation of antioxidant and phenolic data indicated that cultivars with higher ODC and FC values, particularly Sousão and Verdelho, consistently exhibited superior antioxidant performance across all assays, supporting a strong structure–activity relationship.

### 3.3 Identification and Quantification of Phenolic Compounds by HPLC–DAD–ESI–MS/MS

The quantification of phenolic compounds in grape stems revealed substantial diversity among white and red Douro varieties. Table 3 presents the concentrations of individual proanthocyanidins, flavonols, and stilbenes detected, expressed as mean ± standard deviation. The compounds are displayed by class to facilitate interpretation of varietal differences and to highlight the contribution of specific subclasses such as procyanidin oligomers, quercetin derivatives, and stilbene polymers.

**TABLE 3:** Quantification of individual phenolic compounds in grape stem extracts from 12 Douro cultivars determined by HPLC–DAD–ESI–MS/MS.

Class/Compound	Retention time	[M‐H]	Tinta Amarela	Roriz	Sousão	Tinta Francisca	Rufete	Folgasão	Síria	Rabigato	Gouveio	Verdelho	Códega de Larinho	Cercial
			Proanthocyanidins											
Procyanidin tetramer B‐type	1.33	1153	136.36 ± 0.04^a^	128.92 ± 0.04^d^	130.91 ± 0.00^b^	130.39 ± 0.15^b,c^	115.99 ± 0.06^f^	129.57 ± 0.04^c^	128.21 ± 0.02^d^	122.11 ± 0.01^e^	102.55 ± 0.05^i^	102.72 ± 0.04^i^	106.43 ± 0.00^h^	107.68 ± 0.00^g^
Proanthocyanidin dimer (B‐type)	2.15	577	152.33 ± 0.00^d^	176.68 ± 0.10^c^	246.92 ± 0.09^a^	69.24 ± 0.02^h^	226.37 ± 0.01^b^	53.70 ± 0.01^e^	57.88 ± 0.02^k^	71.69 ± 0.07^g^	108.02 ± 0.21^e^	59.86 ± 0.02^j^	64.26 ± 0.00^i^	95.88 ± 0.05^g^
Proanthocyanidin trimer (B‐type)	2.28	865	127.09 ± 0.04 ^d^	123.66 ± 0.04^f^	143.89 ± 0.02^a^	94.23 ± 0.01^i^	53.43 ± 0.00^k^	96.15 ± 0.05^h^	92.66 ± 0.12^j^	94.77 ± 0.04^i^	133.98 ± 0.03^b^	126.87 ± 0.04^e^	128.41 ± 0.00^c^	122.73 ± 0.04^g^
Proanthocyanidin trimer monogallate	2.31	1017	65.01 ± 0.01^i^	97.59 ± 0.26^f^	76.49 ± 0.02^g^	118.98 ± 0.01^e^	72.21 ± 0.01^h^	55.10 ± 0.02^L^	131.13 ± 0.05^d^	134.94 ± 0.05^c^	61.51 ± 0.03^j^	151.18 ± 0.04^a^	145.19 ± 0.00^b^	59.84 ± 0.01^k^
Proanthocyanidin trimer (iso 2)	4.15	865	63.59 ± 0.04^c^	58.53 ± 0.00^h^	59.33 ± 0.00^g^	58.53 ± 0.00^j^	23.35 ± 0.00^e^	56.69 ± 0.05^f^	62.29 ± 0.01^d^	60.46 ± 0.00^e^	52.68 ± 0.01^k^	64.67 ± 0.01^b^	67.83 ± 0.00^a^	56.41 ± 0.04^j^
Proanthocyanidin trimer mono (iso 2)	6.75	1017	11.21 ± 0.00^j^	98.44 ± 0.05^b^	91.74 ± 0.04^c^	58.53 ± 0.04^f^	280.26 ± 0.00^a^	57.57 ± 0.00^g^	54.47 ± 0.00^h^	49.35 ± 0.00^i^	61.38 ± 0.00^d^	58.41 ± 0.01^f^	59.27 ± 0.00^e^	59.58 ± 0.00^e^
			Flavonols											
Rutin	7.14	609	ND	256.22 ± 0.02^a^	27.89 ± 0.01^h^	76.73 ± 0.00^b^	1.05 ± 0.01^k^	15.16 ± 0.01^i^	58.49 ± 0.01^c^	57.51 ± 0.01^d^	54.79 ± 0.01^f^	29.21 ± 0.00^g^	56.81 ± 0.00^g^	14.82 ± 0.00^j^
Quercetin‐3‐O‐glucuronide	7.45	477	ND	244.75 ± 0.03^e^	225.25 ± 0.02^f^	24.72 ± 0.04	ND	29.95 ± 0.06^h^	379.97 ± 0.09^c^	387.65 ± 0.02^b^	370.00 ± 0.00^d^	30.25 ± 0.00^h^	85.99 ± 0.00^g^	433.73 ± 0.00^a^
Quercetin 3‐galactoside	8.07	463	19.09 ± 0.58^f^	35.74 ± 0.58^d^	14.33 ± 0.00^h^	46.44 ± 0.00^c^	ND	ND	360.99 ± 0.09^a^	17.88 ± 0.03^g^	24.75 ± 0.01^e^	24.93 ± 0.01^e^	56.59 ± 0.05^b^	ND
Quercetin	11.08	301	ND	54.89 ± 0.09^b^	25.60 ± 0.04^d^	11.96 ± 0.00^f^	29.01 ± 0.45^c^	24.94 ± 0.02^e^	ND	1.25 ± 0.00	ND	157.13 ± 0.00^a^	6.36 ± 0.00	4.32 ± 0.01
			Stilbenes											
Viniferin	12.68	453	340.23 ± 0.00^d^	111.41 ± 0.01^h^	190.84 ± 0.01^g^	112.45 ± 0.00^f^	217.73 ± 0.04^e^	207.38 ± 0.00^f^	423.77 ± 0.09^b^	467.44 ± 0.05^a^	ND	109.18 ± 0.01^i^	85.99 ± 0.00^j^	405.49 ± 0.07^c^
Resveratrol tetramer iso 1	13.43	905	ND	292.91 ± 0.00^d^	343.35 ± 0.17^b^	363.47 ± 0.19^a^	6.52 ± 0.00^g^	ND	4.07 ± 0.00^h^	36.16 ± 0.02^f^	337.44 ± 0.15^c^	ND	42.22 ± 0.00^e^	6.55 ± 0.02
Resveratrol tetramer iso 2	15.05	905	ND	ND	43.12 ± 0.08^b^	42.16 ± 0.08^c^	5.33 ± 0.01^g^	ND	105.78 ± 0.06^a^	14.32 ± 0.01^e^	39.27 ± 0.06^d^	ND	ND	10.87 ± 0.02^f^

*Note:* Results are expressed as mean ± standard deviation (mg/g dw, *n* = 3). Different letters within each row indicate statistically significant differences among cultivars (*p* < 0.05), according to one‐way ANOVA followed by Tukey's post hoc test.

Abbreviations: dw, dry weight; HPLC–DAD–ESI‐MS/MS, high‐performance liquid chromatography with diode array detection and electrospray ionization tandem mass spectrometry.

In white grape stems, proanthocyanidin derivatives were predominant, particularly the procyanidin tetramer B‐type, with values ranging from 102.55 mg/g in Rabigato to 136.36 mg/g in Tinta Amarela, these differences being statistically significant (*p* < 0.05). Similarly, proanthocyanidin trimers showed a strong varietal effect: Gouveio (133.98 ± 0.03 mg/g) and Verdelho (126.87 ± 0.04 mg/g) displayed significantly higher concentrations than Folgasão and Rufete (< 100 mg/g). The trimer monogallate (isomer 1) was particularly abundant in Verdelho (151.18 ± 0.04 mg/g), significantly differing from all other varieties. These results confirm that oligomeric proanthocyanidins are the main phenolic fraction of white grape stems, though with marked varietal variation.

In the flavonol group, the varietal effect was even more evident. Rutin was detected in all samples but with significantly higher levels in Roriz (256.22 ± 0.02 mg/g) and residual amounts in Cercial (14.82 ± 0.00 mg/g). Quercetin‐3‐*O*‐glucuronide varied drastically among cultivars, with Cercial (433.73 ± 0.00 mg/g) showing much higher levels than Gouveio (370.00 ± 0.00 mg/g) and Rabigato (379.97 ± 0.09 mg/g), while Folgasão and Tinta Amarela showed no detection. Quercetin 3‐*O*‐galactoside also exhibited cultivar‐specific accumulation, with Síria (360.99 ± 0.09 mg/g) being significantly richer than all other varieties. Free quercetin was highest in Verdelho (157.13 mg/g), significantly differing from all remaining cultivars.

Among stilbenes, ε‐viniferin reached notably high levels in Rabigato (423.77 ± 0.09 mg/g) and Cercial (405.49 ± 0.07 mg/g), significantly differing from the other varieties, where values were lower or even undetected (e.g., Gouveio). Resveratrol tetramers also showed a cultivar‐dependent pattern: isomer 1 was higher in Tinta Francisca (363.47 ± 0.19 mg/g) and Gouveio (337.44 ± 0.15 mg/g), while isomer 2 stood out in Síria (105.78 ± 0.06 mg/g), significantly higher than in Cercial and Rufete (< 15 mg/g).

In red grape stems, statistical differences were also marked. Proanthocyanidin dimer B‐type reached its highest value in Sousão (246.92 ± 0.09 mg/g), significantly differing from Roriz (176.68 ± 0.10 mg/g) and Tinta Amarela (152.33 ± 0.00 mg/g). The procyanidin tetramer was most abundant in Tinta Amarela (136.36 ± 0.04 mg/g), while the trimer monogallate (isomer 2) was extraordinarily high in Rufete (280.26 ± 0.00 mg/g), clearly differing from all other cultivars (< 100 mg/g).

In the flavonol fraction of red stems, only a few cultivars stood out: Roriz exhibited the highest levels of rutin (256.22 ± 0.02 mg/g, letter a), quercetin‐3‐*O*‐glucuronide (244.75 ± 0.03 mg/g), and free quercetin (54.89 ± 0.09 mg/g), significantly differing from the other cultivars. Tinta Francisca was the main source of quercetin 3‐*O*‐galactoside (46.44 ± 0.00 mg/g).

Regarding stilbenes, Tinta Amarela presented the highest levels of ε‐viniferin (340.23 ± 0.00 mg/g), while Tinta Francisca was the richest in resveratrol tetramers (343.35 ± 0.17 mg/g for isomer 1). These values significantly differed from cultivars such as Rufete and Sousão, which showed lower concentrations.

Overall, the results confirm that grape stems, regardless of berry color, are an important source of oligomeric proanthocyanidins, flavonols, and stilbenes, but the quantitative profiles show statistically significant differences between cultivars. White varieties, particularly Cercial, Rabigato, and Gouveio, stand out for their accumulation of flavonols (quercetin and derivatives), while red cultivars such as Sousão, Rufete, and Roriz are richer in proanthocyanidins and resveratrol tetramers. These differences, already described in previous studies [38, 44], reinforce the importance of grape cultivar choice as a determinant factor for the bioactive valorization of grape stems.

When individual phenolic profiles obtained by HPLC–DAD–ESI–MS/MS were jointly interpreted with antioxidant capacity and enzyme inhibition data, clear structure–activity relationships emerged. Cultivars enriched in oligomeric proanthocyanidins, particularly procyanidin dimers, trimers, and tetramers (e.g., Sousão, Rufete, and Verdelho), consistently exhibited higher antioxidant capacity and stronger inhibition of elastase and hyaluronidase. In contrast, cultivars characterized by elevated levels of flavonols and stilbene derivatives, such as quercetin conjugates and ε‐viniferin (e.g., Cercial, Rabigato, and Gouveio), showed a more pronounced association with tyrosinase inhibition and depigmenting activity.

Although the present study provides a comprehensive quantification of phenolic subclasses using HPLC–DAD, future work should integrate mass spectrometry techniques (LC–MS/MS or QTOF‐MS) to unequivocally confirm compound identity, especially in the case of oligomeric proanthocyanidins and resveratrol tetramers, where co‐elution or overlapping UV spectra may lead to misassignments. Such approaches would further strengthen the comparison with existing literature data, which increasingly rely on MS‐based characterization.

These chemical fingerprints help to explain the multifunctional and selective bioactivity patterns discussed in Section 3.4.

### 3.4 Anti‐Aging and Skin Depigmenting Activity

To corroborate whether the phenolic extracts studied had anti‐aging and depigmenting effects, the inhibitory activity of the different grape stems samples on three key enzymes involved in physiological and cosmetic processes namely elastase, hyaluronidase, and tyrosinase was analyzed. The results were expressed as mean inhibition percentage activity ± standard deviation in Table 4.

**TABLE 4:** Anti‐elastase, anti‐hyaluronidase, and anti‐tyrosinase activities of grape stem extracts at 1 mg/mL.

Grape stem	% Inhibition elastase	% Inhibition hyaluronidase	% Inhibition tyrosinase
Tinta Amarela	66.35 ± 1.34^ab^	49.69 ± 4.75^cd^	55.60 ± 0.63^a^
Tinta Roriz	60.20 ± 1.54^d^	55.35 ± 4.75^bc^	47.36 ± 2.29^b^
Sousao	60.64 ± 2.19^cd^	63.52 ± 1.09^ab^	58.14 ± 1.10^a^
Tinta Francisca	65.99 ± 1.09^abc^	41.51 ± 3.77^c^	56.03 ± 1.60^a^
Rufeta	65.85 ± 2.23^abc^	59.75 ± 5.76^abc^	56.03 ± 1.60^a^
Folgasao	63.02 ± 1.20^abcd^	60.38 ± 4.99^abc^	53.28 ± 7.13^b^
Siria	63.39 ± 1.75^abcd^	58.49 ± 1.89^abc^	55.39 ± 1.83^a^
Rabigato	64.11 ± 1.23^abcd^	64.15 ± 6.54^ab^	55.81 ± 0.97^a^
Gouveio	67.15 ± 2.38^a^	67.92 ± 6.80^ab^	58.35 ± 3.00^a^
verdelho	62.74 ± 2.52^abcd^	69.81 ± 5.66^a^	60.68 ± 1.10^a^
Codega do larinho	58.97 ± 1.70^d^	47.80 ± 1.09^cd^	54.12 ± 1.46^b^
Cercial	61.58 ± 2.64^bcd^	66.04 ± 3.77^ab^	56.66 ± 0.97^a^

*Note:* Data is presented as median with range (*n* = 3). Different letters within the same column indicate statistically significant differences among cultivars (*p* < 0.05), according to Tukey's test followed by Dunn's post hoc test.

Regarding elastase inhibition, all extracts showed inhibitory activity above 58%, with values ranging from 58.97% ± 1.70% to 67.15% ± 2.38%. Sample 9 (Gouveio) exhibited the highest inhibition (67.15%), followed closely by sample 1 (Tinta Amarela, 66.35% ± 1.34%), both differing significantly from the least active samples. In contrast, samples 2 (Tinta Roriz) and 11 (Códega de Larinho) displayed the lowest elastase inhibition, suggesting a reduced capacity to modulate collagen‐degrading enzymes.

Hyaluronidase inhibition showed a wider variability, with values ranging from 41.51% ± 3.77% to 69.81% ± 5.66%. Sample 10 (Verdelho) presented the highest inhibitory activity (69.81%), followed by samples 9 (Gouveio) and 12 (Cercial), all exceeding 66%. Conversely, samples 1, 4, and 11 exhibited significantly lower inhibition, indicating limited efficacy against hyaluronic acid degradation.

Tyrosinase inhibition values ranged between 47.36% ± 2.29% and 60.68% ± 1.10%. Sample 10 again showed the highest inhibition (60.68%), followed by samples 9 and 3 (Sousão), all exceeding 58%. Sample 2 displayed the lowest tyrosinase inhibition, significantly differing from most cultivars, which reflects its lower depigmenting potential.

When enzyme inhibition data were jointly interpreted alongside phenolic composition and antioxidant capacity, certain cultivars exhibited multifunctional bioactivity profiles. In particular, Gouveio, Verdelho, and Sousão simultaneously showed high antioxidant capacity and strong inhibition of elastase, hyaluronidase, and tyrosinase, highlighting their broad‐spectrum cosmetic potential. These cultivars consistently ranked among the most active extracts across all biological assays, suggesting a synergistic contribution of multiple phenolic subclasses.

Conversely, other cultivars displayed more selective enzymatic profiles. Samples 5, 6, 7, 8, and 12 showed high inhibitory activity against two of the three enzymes but did not consistently perform across all targets, indicating enzyme‐specific mechanisms of action. In contrast, samples 2 and 11 generally exhibited lower inhibitory activity, particularly against elastase and tyrosinase, reflecting their reduced phenolic diversity and bioactivity potential.

**FIGURE 1:**
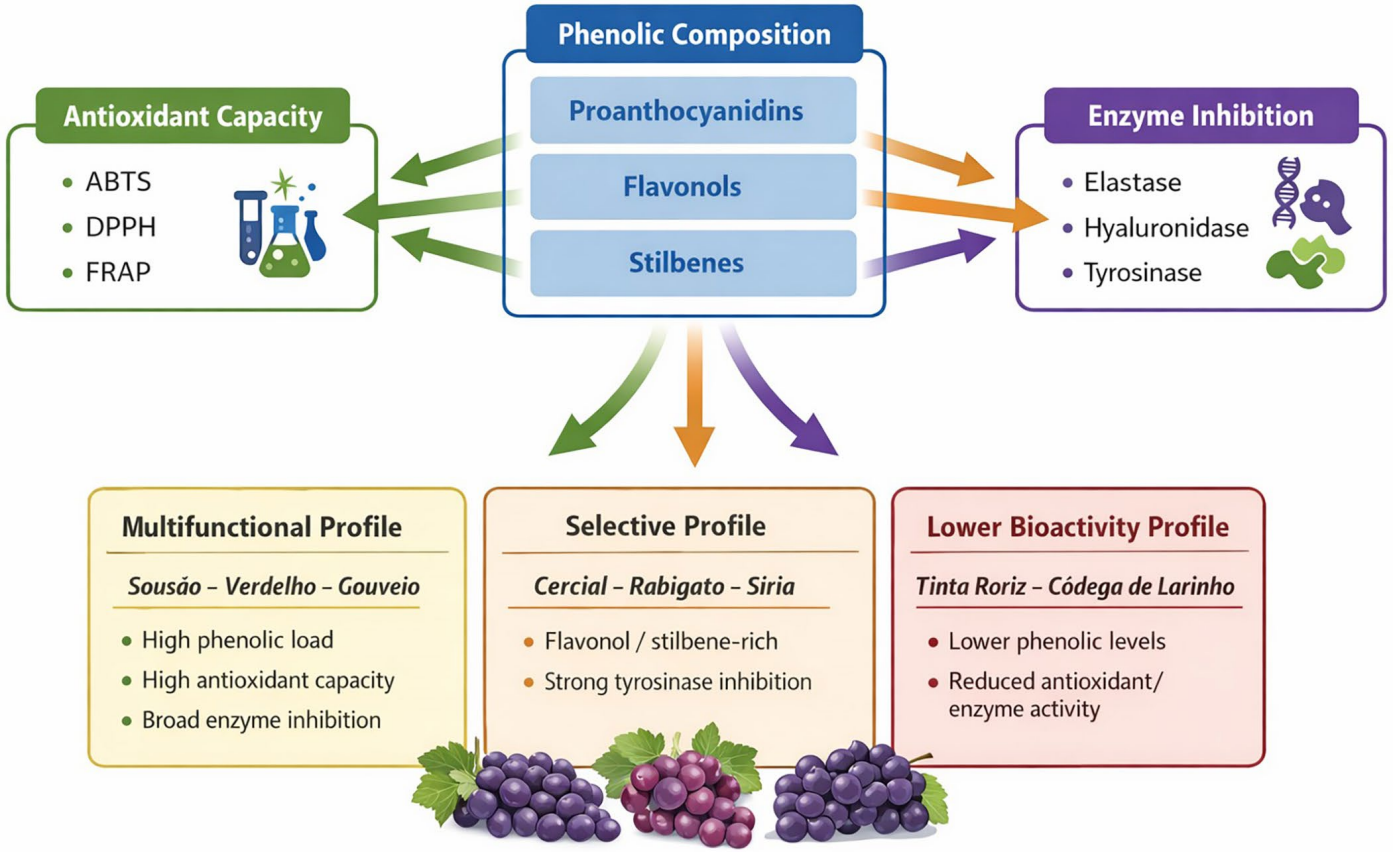
Schematic representation of the integrated relationship between phenolic composition, antioxidant capacity, and enzyme inhibitory activity of grape stem extracts, highlighting multifunctional and selective bioactivity profiles.

An integrated overview of the relationships between phenolic composition, antioxidant capacity, and enzyme inhibitory activity is schematically illustrated in Figure 1, which summarizes the functional bioactivity profiles observed among the studied cultivars and highlights their potential for targeted or multifunctional cosmetic applications.

These findings are consistent with previous reports demonstrating that phenolic‐rich plant extracts exert inhibitory effects against elastase, hyaluronidase, and tyrosinase through complementary mechanisms [26, 27, 30]. Although in vitro assays provide valuable mechanistic insights, further validation using cellular models and formulation‐based studies is required to confirm bioavailability, stability, and efficacy in dermocosmetic applications. Nonetheless, the present results reinforce the potential of grape stem extracts as multifunctional bioactive ingredients, supporting their valorization within sustainable cosmetic and nutraceutical frameworks.

### 3.5 Correlation Between Phenolic Composition and Enzyme Inhibition Activities

The inhibitory activities against elastase, hyaluronidase, and tyrosinase varied significantly among the extracts, indicating that the phenolic composition strongly modulates their bioactivity.

For elastase inhibition, the most effective samples were 9 (67.15%) and 1 (66.35%), which also corresponded to extracts particularly rich in proanthocyanidin dimers and trimers. This observation is consistent with previous studies reporting that condensed tannins interact with the enzyme's active site through hydrogen bonding and hydrophobic interactions, leading to strong anti‐elastase effects [27, 30]. The abundance of procyanidin tetramers (B‐type) in both white and red varieties, particularly in Sousão and Rufete, likely contributed to the observed inhibition.

Regarding hyaluronidase, the highest activity was observed in samples 10 (69.81%) and 9 (67.92%), which were characterized by elevated levels of stilbenes, including ε‐viniferin and resveratrol tetramers. Stilbene oligomers have been widely described as potent modulators of hyaluronidase activity, mainly due to their ability to chelate catalytic residues and block the enzymatic mechanism [30, 31]. In addition, the high concentrations of quercetin‐3‐O‐glucuronide in certain white varieties (e.g., Cercial and Rabigato) may have enhanced the inhibitory potential, since quercetin derivatives are well‐established hyaluronidase inhibitors [29].

For tyrosinase inhibition, the most active extracts were samples 10 (60.68%), 9 (58.35%), and 3 (58.14%), all exceeding 55%. These extracts were particularly rich in quercetin glycosides (notably quercetin‐3‐galactoside and rutin) and stilbene tetramers. The inhibitory capacity of flavonols, especially quercetin derivatives, has been attributed to their structural similarity to L‐DOPA, which allows them to act as competitive inhibitors of tyrosinase [27, 29]. The high activity observed in sample 10 likely results from the synergistic contribution of quercetin glycosides and viniferin derivatives.

Extracts with lower phenolic diversity, such as sample 11, showed the weakest activities across all enzymes (58.97% elastase, 47.80% hyaluronidase, 54.12% tyrosinase). This reinforces the concept that bioactivity is driven by the combined contribution of different phenolic classes rather than the dominance of a single compound, as previously emphasized in natural product research [32, 33].

Overall, these results highlight a clear correlation between phenolic composition and enzyme inhibition: proanthocyanidins were strongly associated with elastase inhibition, stilbenes with hyaluronidase modulation, and flavonols with tyrosinase inhibition. While these findings underline the multifunctional potential of grape stem extracts for cosmetic and nutraceutical applications, further in vivo validation and stability assessments are required to substantiate their applicability in complex biological systems [28, 31].

## 4 Conclusions

This study demonstrates that grape stem extracts from Douro cultivars are valuable sources of phenolic compounds with relevant antioxidant, anti‐aging, and depigmenting activities. Significant cultivar‐dependent differences were observed in phenolic composition and bioactivity, highlighting the importance of varietal selection for dermocosmetic applications. Among the 12 cultivars analyzed, Sousão, Verdelho, and Gouveio consistently exhibited the most pronounced multifunctional activity, with inhibition levels above 60% for elastase, hyaluronidase, and tyrosinase, enzymes directly involved in skin aging, extracellular matrix degradation, and pigmentation processes.

The strong bioactivity of these cultivars is associated with their enrichment in oligomeric proanthocyanidins, stilbene derivatives (including ε‐viniferin and resveratrol tetramers), and flavonols such as quercetin derivatives, suggesting a synergistic contribution of multiple phenolic classes. Notably, white cultivars such as Verdelho and Cercial showed particularly high flavonol contents, supporting their potential use in cosmetic formulations aimed at skin brightening and protection against oxidative stress. In contrast, red cultivars, including Sousão and Rufete, were characterized by higher levels of proanthocyanidins and stilbenes, consistent with their antioxidant and anti‐elastase activities relevant to anti‐wrinkle strategies.

Overall, these findings support the valorization of grape stems as sustainable, high‐value ingredients for cosmetic and dermatological applications, in line with circular economy principles. While the present results are based on in vitro assays, further studies addressing bioavailability, stability, safety, and efficacy in cellular and formulation‐based models are required to confirm their applicability in dermocosmetic products. Nevertheless, this work provides a robust scientific basis for the targeted selection of grape cultivars and the development of multifunctional cosmetic ingredients derived from winery by‐products.

## Author Contributions

**Maria Garcia‐Marti:** conceptualization, data curation, formal analysis, investigation, methodology, writing – original draft, writing – review and editing. **Oumaima Boutaub:** methodology, writing – review and editing. **Amanda Priscila Silva Nascimento:** data curation, methodology, writing – review and editing. **Jesus Simal‐Gandara:** writing – review and editing. **Ana Novo Barros:** funding acquisition, project administration, supervision, writing – original draft, writing – review and editing.

## Funding

This work was supported by National Funds through FCT–the Portuguese Foundation for Science and Technology under the projects UID/04033/2025–Centre for the Research and Technology of Agro‐Environmental and Biological Sciences, LA/P/0126/2020.

## Conflicts of Interest

The authors declare no conflicts of interest.

## Data Availability

The data that support the findings of this study are available on request from the corresponding author. The data are not publicly available due to privacy or ethical restrictions.

## References

[1] O. E. Constantin , F. Stoica , R. N. Rațu , N. Stănciuc , G. E. Bahrim , and G. Râpeanu , “Bioactive Components, Applications, Extractions, and Health Benefits of Winery By‐Products From a Circular Bioeconomy Perspective: A Review,” Antioxidants 13, no. 1 (2024): 100, 10.3390/antiox13010100. 38247524 PMC10812587

[2] R. Ferrer‐Gallego and P. Silva , “The Wine Industry By‐Products: Applications for Food Industry and Health Benefits,” Antioxidants 11, no. 10 (2022): 2025, 10.3390/antiox11102025. 36290748 PMC9598427

[3] OIV , World Wine Production Outlook – OIV First Estimates (November 2023, pp. 1–9) (International Organisation of Vine and Wine, 2023), https://www.oiv.int/public/medias/8553/en‐oiv‐2021‐world‐wine‐production‐first‐estimates‐to‐update.pdf.

[4] M. del Mar Contreras , J. M. Romero‐García , J. C. López‐Linares , I. Romero , and E. Castro , “Residues From Grapevine and Wine Production as Feedstock for a Biorefinery,” Food and Bioproducts Processing 134 (2022): 56–79, 10.1016/j.fbp.2022.05.005.

[5] V.‐C. Niculescu and R.‐E. Ionete , “An Overview on Management and Valorisation of Winery Wastes,” Applied Sciences 13, no. 8 (2023): 5063, 10.3390/app13085063.

[6] A. K. Chakka and A. S. Babu , “Bioactive Compounds of Winery By‐Products: Extraction Techniques and Their Potential Health Benefits,” Applied Food Research 2, no. 1 (2022): 100058, 10.1016/j.afres.2022.100058.

[7] P. A. Onache , E.‐I. Geana , C. T. Ciucure , et al., “Bioactive Phytochemical Composition of Grape Pomace Resulted From Different White and Red Grape Cultivars,” Separations 9, no. 12 (2022): 395, 10.3390/separations9120395.

[8] M. Guaita , L. Panero , S. Motta , B. Mangione , and A. Bosso , “Effects of High‐Temperature Drying on the Polyphenolic Composition of Skins and Seeds From Red Grape Pomace,” LWT 145 (2021): 111323, 10.1016/j.lwt.2021.111323.

[9] A. Tikhonova , N. Ageeva , and E. Globa , “Grape Pomace as a Promising Source of Biologically Valuable Components,” BIO Web of Conferences, 34, 6002, (2021), 10.1051/bioconf/20213406002.

[10] A. R. Machado , T. Atatoprak , J. Santos , et al., “Potentialities of the Extraction Technologies and Use of Bioactive Compounds From Winery By‐Products: A Review From a Circular Bioeconomy Perspective,” Applied Sciences 13, no. 13 (2023): 7754, 10.3390/app13137754.

[11] A. Silva , V. Silva , G. Igrejas , et al., “Phenolic Compounds Classification and Their Distribution in Winemaking By‐Products,” European Food Research and Technology 249, no. 2 (2023): 207–239, 10.1007/s00217-022-04163-z.

[12] A. Chetrariu , A. Dabija , L. Caisin , V. Agapii , and I. Avrămia , “Sustainable Valorization of Wine Lees: From Waste to Value‐Added Products,” Applied Sciences 15, no. 7 (2025): 3648, 10.3390/app15073648.

[13] M. B. S. Gonçalves , M. P. Marques , F. Correia , et al., “Wine Industry By‐Products as a Source of Active Ingredients for Topical Applications,” Phytochemistry Reviews 24, no. 5 (2024): 1, 10.1007/s11101-024-10030-4.

[14] O. I. Tsiapali , E. Ayfantopoulou , A. Tzourouni , A. Ofrydopoulou , S. Letsiou , and A. Tsoupras , “Unveiling the Utilization of Grape and Winery by‐Products in Cosmetics With Health Promoting Properties,” Applied Sciences 15, no. 3 (2025): 1007, 10.3390/app15031007.

[15] S. Ferreyra , R. Bottini , and A. Fontana , “Background and Perspectives on the Utilization of Canes' and Bunch Stems' Residues From Wine Industry as Sources of Bioactive Phenolic Compounds,” Journal of Agricultural and Food Chemistry 71, no. 23 (2023): 8699–8730, 10.1021/acs.jafc.3c01635. 37267502

[16] P. Goufo , R. K. Singh , and I. Cortez , “A Reference List of Phenolic Compounds (Including Stilbenes) in Grapevine (*Vitis vinifera* L.) Roots, Woods, Canes, Stems, and Leaves,” Antioxidants 9, no. 5 (2020): 398, 10.3390/antiox9050398. 32397203 PMC7278806

[17] R. Mangione , R. Simões , H. Pereira , et al., “Potential Use of Grape Stems and Pomaces From Two Red Grapevine Cultivars as Source of Oligosaccharides,” Processes 10, no. 9 (2022): 1896, 10.3390/pr10091896.

[18] M. A. I. Maamoun , “An Insight Into the Brilliant Benefits of Grape Waste,” in Mediterranean Fruits Bio‐Wastes: Chemistry, Functionality and Technological Applications (Springer, 2022), 433–465, 10.1007/978-3-030-84436-3_18.

[19] F. B. Dilek , D. San Martin , M. Gutierrez , J. Ibarruri , B. Iñarra , and U. Yetis , “Assessing Environmental and Economic Sustainability: Valorizing Grape Stems for Animal Feed Production,” ACS Sustainable Chemistry & Engineering 12, no. 50 (2024): 18028–18042, 10.1021/acssuschemeng.4c06005. 39703410 PMC11653404

[20] N. van Wyk , C. Borgmeier , A. Kleber , and E. M. Gabor , Sustainable Approaches in Viticulture: From Wastes and Side Streams to High‐Value Products (Springer, 2025), 10.1007/10_2025_281. 40251458

[21] R. Fernandes , C. Medrano‐Padial , R. Dias‐Costa , et al., “Grape Stems as Sources of Tryptophan and Selenium: Functional Properties and Antioxidant Potential,” Food Chemistry: X 26 (2025): 102260, 10.1016/j.fochx.2025.102260. 39995406 PMC11848443

[22] A. S. G. Costa , R. C. Alves , A. F. Vinha , M. Barros , and M. B. P. P. Oliveira , “Influence of Variety and Harvest Year on Phenolic Composition and Antioxidant Capacity of Grape Stems,” Industrial Crops and Products 195 (2023): 116478, 10.1016/j.indcrop.2023.116478.

[23] C. Costa , J. Campos , I. Gouvinhas , A. R. Pinto , M. J. Saavedra , and A. N. Barros , “Unveiling the Potential of Unexplored Winery By‐Products From the Dão Region: Phenolic Composition, Antioxidants, and Antimicrobial Properties,” Applied Sciences 13, no. 18 (2023): 10020, 10.3390/app131810020.

[24] A. T. Serra , A. A. Matias , A. V. M. Nunes , et al., “Grape Stems as a Source of Phenolic Compounds: Optimization of the Extraction Process and Evaluation of Their Antioxidant Activity,” Antioxidants 12 (2023): 1165, 10.3390/antiox12061165. 37371895 PMC10294785

[25] M. Serra , A. Casas , J. A. Teixeira , and A. N. Barros , “Revealing the Beauty Potential of Grape Stems: Harnessing Phenolic Compounds for Cosmetics,” International Journal of Molecular Sciences 24, no. 14 (2023): 11751, 10.3390/ijms241411751. 37511513 PMC10380576

[26] M. del Carmen Villegas‐Aguilar , M. de la Luz Cádiz‐Gurrea , D. Arráez‐Román , and A. Segura‐Carretero , “Anti‐Aging Effects of Phenolic Compounds,” in Anti‐Aging Pharmacology (Elsevier, 2023), 119–152, 10.1016/B978-0-12-823679-6.00017-5.

[27] J. M. Andrade , E. M. Domínguez‐Martín , M. Nicolai , C. Faustino , L. M. Rodrigues , and P. Rijo , “Screening the Dermatological Potential of *Plectranthus* Species Components: Antioxidant and Inhibitory Capacities Over Elastase, Collagenase and Tyrosinase,” Journal of Enzyme Inhibition and Medicinal Chemistry 36, no. 1 (2021): 258–270, 10.1080/14756366.2020.1862099. PMC7808741 33322969

[28] F. Papaccio , A. D'Arino , S. Caputo , and B. Bellei , “Focus on the Contribution of Oxidative Stress in Skin Aging,” Antioxidants 11, no. 6 (2022): 1121, 10.3390/antiox11061121. 35740018 PMC9220264

[29] H.‐M. Liu , M.‐Y. Cheng , M.‐H. Xun , et al., “Possible Mechanisms of Oxidative Stress‐Induced Skin Cellular Senescence, Inflammation, and cancer and the Therapeutic Potential of Plant Polyphenols,” International Journal of Molecular Sciences 24, no. 4 (2023): 3755, 10.3390/ijms24043755. 36835162 PMC9962998

[30] C. D. Papaemmanouil , J. Peña‐García , A. J. Banegas‐Luna , et al., “ANTIAGE‐DB: A Database and Server for the Prediction of Anti‐Aging Compounds Targeting Elastase, Hyaluronidase, and Tyrosinase,” Antioxidants 11, no. 11 (2022): 2268, 10.3390/antiox11112268. 36421454 PMC9686885

[31] N. Bharadvaja , S. Gautam , and H. Singh , “Natural Polyphenols: A Promising Bioactive Compounds for Skin Care and Cosmetics,” Molecular Biology Reports 50, no. 2 (2023): 1817–1828, 10.1007/s11033-022-08156-9. 36494596

[32] G. Bjørklund , M. Shanaida , R. Lysiuk , et al., “Natural Compounds and Products From an Anti‐Aging Perspective,” Molecules 27, no. 20 (2022): 7084, 10.3390/molecules27207084. 36296673 PMC9610014

[33] E. Csekes and L. Račková , “Skin Aging, Cellular Senescence and Natural Polyphenols,” International Journal of Molecular Sciences 22, no. 23 (2021): 12641, 10.3390/ijms222312641. 34884444 PMC8657738

[34] C. Breda , A. Nascimento , P. Meghwar , et al., “Phenolic Composition and Antioxidant Activity of Edible Flowers: Insights From Synergistic Effects and Multivariate Analysis,” Antioxidants 14, no. 3 (2025): 282, 10.3390/antiox14030282. 40227247 PMC11939731

[35] Z. Branco , F. Baptista , J. Paié‐Ribeiro , I. Gouvinhas , and A. N. Barros , “Impact of Winemaking Techniques on the Phenolic Composition and Antioxidant Properties of Touriga Nacional Wines,” Molecules 30, no. 7 (2025): 1601, 10.3390/molecules30071601. 40286197 PMC11990232

[36] S. Vieira , A. Abraão , M. Arranca , et al., “Fostering Sustainability: Enzymatic Modeling of Juice Production Pomace for Advancing Innovative Ingredients in Non‐Dairy Infant Food Applications,” Food Chemistry 493 (2025): 145962, 10.1016/j.foodchem.2025.145962. 40816070

[37] M. Serra , C. Botelho , H. Almeida , A. Casas , J. A. Teixeira , and A. N. Barros , “Stable and Functional Cosmetic Creams Enriched With Grape Stem Extract: A Sustainable Skincare Strategy,” Antioxidants 14, no. 7 (2025): 784, 10.3390/antiox14070784. 40722888 PMC12291674

[38] I. Esparza , J. Moler , M. Arteta , N. Jiménez‐Moreno , and C. Ancín‐Azpilicueta , “Phenolic Composition of Grape Stems From Different Spanish Varieties and Vintages,” Biomolecules 11 (2021): 1221, 10.3390/biom11081221. 34439886 PMC8392641

[39] C. Leal , R. Santos , R. Pinto , et al., “Recovery of Bioactive Compounds From White Grape (*Vitis vinifera* L.) Stems as Potential Antimicrobial Agents for Human Health,” Saudi Journal of Biological Sciences 27 (2020): 1009–1015, 10.1016/j.sjbs.2020.02.013. 32256161 PMC7105666

[40] Y. El Rayess , N. Nehme , S. Azzi‐Achkouty , and S. G. Julien , “Wine Phenolic Compounds: Chemistry, Functionality and Health Benefits,” Antioxidants 13 (2024): 1312, 10.3390/antiox13111312. 39594454 PMC11591289

[41] J. Leal , A. Costa , and F. M. Nunes , “Comparative Study of White Grape Stems as Sources of Phenolics: Impact of Variety and Drying,” Journal of the Science of Food and Agriculture 103 (2023): 5183–5191, 10.1002/jsfa.12967.36882903

[42] J. Portu , I. López‐Alfaro , S. Gómez‐Alonso , R. López , and T. Garde‐Cerdán , “Phenolic Composition of Grape Stems of Tempranillo and Graciano Varieties: Influence of Vineyard and Vintage,” Journal of Food Composition and Analysis 111 (2022): 104653, 10.1016/j.jfca.2022.104653.

[43] C. de Blas , D. R. Figueira , A. M. S. Silva , M. R. Domingues , and A. Vilela , “Valorization of Grape Stems Through Sustainable Extraction: Phytochemical Characterization and Antioxidant Potential,” ACS Sustainable Chemistry & Engineering 12 (2024): 3602–3614, 10.1021/acssuschemeng.4c06005.

[44] C. Negro , A. Aprile , A. Luvisi , L. De Bellis , and A. Miceli , “Antioxidant and Phenolic Profile of Grape Stems From Southern Italian Cultivars: Potential By‐Products for Food Industry,” Molecules 27 (2022): 5679, 10.3390/molecules27175679. 36080445 PMC9457659

[45] M. G. Blackford , D. M. Pereira , M. F. L. Lemos , and I. C. F. R. Ferreira , “Methodological Consistency in Antioxidant Assays: Comparative Assessment of Grape Stem Extracts,” Antioxidants 11 (2022): 1221, 10.3390/antiox11061221.35883713

[46] L. Gaspar , S. Martins , M. Silva , J. M. Cruz , and J. A. Teixeira , “Phenolic Composition and Antioxidant Activity of White Grape Stems: A Comparative Analysis,” Food Bioscience 52 (2023): 102382, 10.1016/j.fbio.2023.102382.

[47] N. Martins and I. C. F. R. Ferreira , “Emerging Uses of White Grape By‐Products in Food and Health: An Updated Review,” Antioxidants 12 (2023): 5437, 10.3390/antiox12275437.

